# Comparative mapping of expressed sequence tags containing microsatellites in rainbow trout (*Oncorhynchus mykiss*)

**DOI:** 10.1186/1471-2164-6-54

**Published:** 2005-04-18

**Authors:** Caird E Rexroad, Maria F Rodriguez, Issa Coulibaly, Karim Gharbi, Roy G Danzmann, Jenefer DeKoning, Ruth Phillips, Yniv Palti

**Affiliations:** 1 USDA/ARS National Center for Cool and Cold Water Aquaculture, Kearneysville, West Virginia 25430 USA; 2 Department of Zoology, University of Guelph, Guelph, Ontario N1G 2W1 Canada; 3 School of Biological Sciences, Washington State University-Vancouver, Vancouver, WA 98686 USA

## Abstract

**Background:**

Comparative genomics, through the integration of genetic maps from species of interest with whole genome sequences of other species, will facilitate the identification of genes affecting phenotypes of interest. The development of microsatellite markers from expressed sequence tags will serve to increase marker densities on current salmonid genetic maps and initiate *in silico *comparative maps with species whose genomes have been fully sequenced.

**Results:**

Eighty-nine polymorphic microsatellite markers were generated for rainbow trout of which at least 74 amplify in other salmonids. Fifty-five have been associated with functional annotation and 30 were mapped on existing genetic maps. Homologous sequences were identified for 20 of the EST containing microsatellites to identify comparative assignments within the tetraodon, mouse, and/or human genomes.

**Conclusion:**

The addition of microsatellite markers constructed from expressed sequence tag data will facilitate the development of high-density genetic maps for rainbow trout and comparative maps with other salmonids and better studied species.

## Background

Genome research in agriculturally important species is facilitated by the availability of species-specific molecular genetic tools and resources such as chromosome maps and large volumes of sequence data. Recently such resources have been developed for important aquaculture species including rainbow trout, which are also widely used as a model system for carcinogenesis, toxicological, and comparative immunological research [1].

Several genetic maps [2-4] consisting primarily of type II markers [5] (amplified fragment length polymorphism simple sequence repeats) have been utilized in the identification of qualitative and quantitative trait loci (QTL) [6] associated with rainbow trout production traits. This includes QTL for natural killer cell-like activity, temperature tolerance, spawning date, body weight, resistance to infectious pancreatic necrosis virus (IPNV), resistance to infectious hematopoietic necrosis virus (IHNV), embryonic development rate, and albinism [7-19]. Although the genetic improvement of these traits through selective breeding would benefit the aquaculture industry, these QTL span large chromosomal intervals and will not be practical for marker assisted selection [20] without additional mapping. The current rainbow trout genetic maps lack marker densities and comparative information necessary to conduct fine mapping aimed at reducing QTL interval sizes, developing practical marker assisted selection schemes, and selection of comparative positional candidates [21] to specifically identify the gene(s) affecting traits of interest.

The recent evolutionary divergence of the salmonids [22] and the importance of many of these species to aquaculture will allow for comparative QTL mapping. For example, the development of genetic linkage maps for Atlantic salmon and Arctic char [23-25] has enabled the identification of QTLs for growth characteristics, disease resistance, and temperature tolerance in those species [18,26,27]. The development of microsatellites markers from EST sequences will facilitate the use of genome information in salmonids species by 1) increasing Type II [5] marker densities on genetic maps; 2) integrating physical and genetic maps; 3) developing comparative genetic maps among salmonids; and 4) developing comparative maps with aquatic model organisms such as zebrafish, fugu, and tetraodon and with better studied avian and mammalian species. This comparative information will aid in the identification of positional candidate genes [28] for production traits in salmonid aquaculture and for basic research which utilizes rainbow trout as model organism.

An expressed sequence tag (EST) [29] project was initiated for rainbow trout with the following aims: 1) identify as many unique transcribed sequences as possible; 2) annotate sequence data with information from other species; 3) develop functional genome tools for rainbow trout; and 4) identify microsatellite and single nucleotide polymorphism (SNP) genetic markers for the construction of high-density chromosome maps [30]. Sequences from a normalized cDNA library (NCCCWA 1RT) constructed from brain, gill, liver, muscle, kidney, and spleen tissue resulted in the creation of the Rainbow Trout Gene Index (RTGI) [31]. Microsatellite marker development was conducted simultaneously with the sequencing phase of the project through hybridization of (GT)_11 _and (GA)_11 _probes to high-density filters representing 27,648 clones from the library. Positive clones were selected for further analyses resulting in 89 polymorphic microsatellite markers derived from ESTs, 30 which were informative in mapping reference families, 55 were associated with functional annotation, and 20 for which comparative mapping assignments were determined.

## Results

### Marker development

Hybridization of high-density filters representing 27,648 cDNA clones from a normalized cDNA library with (GA)_11 _and (GT)_11 _oligonucleotide probes identified 415 clones potentially containing microsatellite repeats. Forward and reverse sequencing for 384 of these clones resulted in 755 sequences of good quality (PHRED score > 20 over 100 bp [32]). Dinucleotide microsatellite repeat were identified from 181 clone sequences. Analysis of redundancy identified 161 unique sequences. PCR primer design was possible for 128 of the 161 sequences which were assigned locus names using OMM5000 nomenclature (in-house terminology for microsatellite markers derived from ESTs). PCR optimization was successful for 93 of the 128 primer pairs. Testing for polymorphism in three reference parents and five doubled haploids resulted in the development of 89 polymorphic microsatellites markers with an average of 4.52 alleles (range 2–7), 40% of which were duplicated as determined by the observance of multiple alleles in clonal lines (see Additional File 1). Cross-amplification in other salmonid species using PCR conditions that were optimized for rainbow trout was determined (Table 1) to be similar to markers from previous publications [33].

**Table 1:** Cross-species amplification. Cross-species amplification allele size range information (bp) for microsatellite markers generated from rainbow trout ESTs

**Locus**	**Artic Char**	**Brook Trout**	**Atlantic Salmon**	**Brown Trout**	**Chinook Salmon**	**Coho Salmon**	**Sockeye Salmon**	**Cutthroat Trout**
OMM5000	253–255	260–294	256	239–254	250–260	256	251–263	240–252
OMM5001	87	89	87	85	97	89	97–109	123–151
OMM5002	142–155	-	165–265	336–346	274–282	139–151	139–151	277
OMM5003	177	176–179	173	179–183	179	168–184	173–186	176–181
OMM5004	187	189	185–187	187	187	150–164	193	189–193
OMM5005	201–203	195–202	219	196–207	186–187	189	191	200–204
OMM5006	-	225–231	-	205–245	203	207–209	201	211–236
OMM5007	162–167	182–187	181–192	166	170–176	180–199	156–170	147–163
OMM5008	259	247	223–255	253–261	246–95	227–252	232	236–254
OMM5009	247	335–363	-	266	279–283	410	-	303–331
OMM5010	288–334	-	346–370	350–358	305–332	341–346	294–297	362
OMM5011	214–248	217–248	213–248	214–233	224–244	227–247	228–249	224–246
OMM5012	170–188	202–208	186	199–201	174–184	175–190	196–223	169–187
OMM5013	-	107	98	98–111	111–135	96–220	126	102–187
OMM5014	-	-	-	201–208	185–202	181–198	-	230–262
OMM5015	228	228	228	228	228	228	228	228
OMM5016	239	-	239	239	198	249–311	189	231–236
OMM5017	203–234	195–200	188–200	208–256	217–237	190–201	193	184–209
OMM5018	192	215–231	182–199	198	184–192	-	-	186–215
OMM5019	298	256–268	269–335	298–321	269–279	272–275	-	272–282
OMM5020	262	262	262–275	262	256–259	255–258	256–261	261–263
OMM5023	131	131	122	122	130–136	122	122	126–142
OMM5024	198	202	170	194–195	209–230	-	-	212–214
OMM5025	154	152	160	160	186–188	158	164	160
OMM5029	210–214	209–227	192–211	193–215	208	207–213	210	203–228
OMM5030	135–187	135–137	140–187	137–139	129–141	150–164	129–141	141–155
OMM5031	145	143	102–144	129–144	142–155	136	142–144	140
OMM5032	198	-	178	216–218	191–201	159	175–179	179–187
OMM5033	284	-	274–280	225	-	243	254–279	260–295
OMM5034	239–287	264	238–240	238–263	239–275	236–269	236–267	246–269
OMM5037	-	266	262–268	260–293	251	254–281	264–272	260–285
OMM5039	284	280	274–280	268	248–286	243–308	282–286	260
OMM5041	168	185–187	172	170	170–173	183–187	132	132–189
OMM5042	133	140	-	127–137	122	-	-	-
OMM5043	122	122	126	114–128	–	112–114	112–122	112–131
OMM5044	206	199	-	230	242	-	-	199–217
OMM5047	-	-	317–328	245–251	257–258	261	190	257–263
OMM5050	242	247–249	251	240	243	245–247	246–248	251–261
OMM5051	174–192	190	179	201–218	212–214	190–191	-	195–206
OMM5053	134–198	-	-	-	122	248	202	228–237
OMM5054	172–240	171–265	161–240	172–241	171–240	171–265	172–241	162–278
OMM5055	217–219	190–220	190–212	190–212	221	225–243	221	219
OMM5056	254–280	268–299	-	196	186	282–319	-	218–253
OMM5058	-	-	216–219	239–244	198–204	192	231–235	194–209
OMM5059	151	-	157	145–172	134	124–135	121–127	126–136
OMM5060	105–164	165	-	160	164	164	164	105–164
OMM5061	400	354–358	291–293	274–282	275	274	262	274–278
OMM5062	229	225	200	192–212	223–233	212–231	221–241	222–240
OMM5063	-	148–195	207–243	203–237	150–154	237–282	154–182	178–220
OMM5064	272–274	92–110	276–283	274	290–319	283–285	279	95–286
OMM5067	153–186	153–164	185–187	171–185	153–186	153–164	171–192	153–187
OMM5072	160–164	161–167	171	170–187	146–158	158–164	158–167	164–170
OMM5074	306–344	247–260	242	238–244	245–253	232	228–233	241–244
OMM5075	214	206–208	-	189–191	186	192–198	194	208–229
OMM5077	368	372	377	383	373	377–380	365	334–354
OMM5088	174	168	-	153	159–161	153	168	159–174
OMM5089	-	-	134–167	-	154–161	132–140	134–146	-
OMM5090	153	152–255	153	249–255	153–269	249–255	153–255	239–248
OMM5091	276	201–210	178	201–205	221–262	368	223–244	265–283
OMM5092	161	161	186	186	202–208	192–217	-	-
OMM5093	285	285	285	285	285	285	285	285
OMM5099	244	243	228	219–234	278–296	260–268	214–254	213–260
OMM5100	137–185	-	182	138–143	173	-	160–173	167–201
OMM5106	358–388	328–353	260–271	261	306–322	361–395	257–274	273–318
OMM5107	258–264	255	250–254	264	-	254	255	-
OMM5108	265	251	265–271	256	263–292	260–262	251–271	256–267
OMM5109	256	254–256	256–271	260–263	256	254–256	260–262	262–271
OMM5112	194	198	194–218	196	193–202	189–196	189	193–206
OMM5113	-	320–368	-	274–320	-	286–304	-	243–288
OMM5117	135–142	138	142	125–140	138	125–140	138	137–154
OMM5121	230	228–230	156–173	166–176	166–267	178–230	173–175	197–222
OMM5124	271–281	271–277	-	272–273	258	266	269	280
OMM5125	256	262–264	254	250–252	256–277	250–260	256–260	256–260
OMM5126	295–299	286	295–299	286–290	286	286–291	286	286–307
% Amp.	87	83	83	97	94	93	85	94

### Functional annotation

Functional annotations were associated with ESTs by BLAST analyses of the RTGI which previously included EST sequence data for the clones described in this manuscript. The highest scoring matches all had E-values ranging from 0 to 10^-40 ^and percent identities ranging from 91–100 % (see Additional File 2). TIGR gene index annotation for tentative consensus sequences (TCs) includes three levels of significance based on percent identity: matches in the range of 90 to 100% are categorized as "homologues," matches in the range of 70–90% are categorized as "similar," and matches less than 70% are categorized as "weakly similar." Annotation of ESTs in this manuscript resulted in 10 highly significant matches to genome sequences, 8 categorized as homologues, 28 as similar, 9 as weakly similar, and 41 for which no associations were determined Locus or gene symbols from Locus Link [34] or UniProt [35] were added to 8 loci designated as homologues.

### Genetic and comparative mapping

Linkage analyses of 33 informative markers resulted in the assignment of 30 markers to linkage groups (see Additional File 3). Twenty-three markers were informative in the reference families of Sakamoto *et al*. [3] and 7 markers were placed on the map of Nichols *et al*. [2] in addition to 3 which were not included into previous linkage groups (Table 2). Comparisons to zebrafish and fugu databases identified homologous assignments for 16 ESTs each (see Additional File 4 and Additional File 5), however, the chromosomal assignments in these 2 species are not yet available.

**Table 2:** Identification of homologous segments between rainbow trout, human, mouse and tetraodaon chromosomes. Rainbow trout linkage group nomenclature is from Nichols *et al*. (2003a)

**Locus**	**Rainbow Trout Linkage Group**	**Tetraodon (TNI)**	**Human (HSA)**	**Mouse (MMU)**
OMM5000	27	8	19	7
OMM5002		21	6	10
OMM5003	23			
OMM5005	11	2	13	14
OMM5012	23			
OMM5017	20			3
OMM5019	9		17	11
OMM5023	22			
OMM5025	8			
OMM5026	29			
OMM5029	12			
OMM5033	16			
OMM5034	19	8		
OMM5041	12	10	3	3
OMM5045	19	12	12	16
OMM5051	?		2	
OMM5056	?		10	14
OMM5057	9			
OMM5059			13	5
OMM5062	27			
OMM5065	25			
OMM5077	25			X
OMM5088	19			
OMM5090	21			
OMM5093	?		4	5
OMM5099	7	6	8	15
OMM5100	15	19		
OMM5106	14			
OMM5107	22	9		
OMM5108	20			
OMM5109	31			
OMM5112	23			
OMM5113			6	10
OMM5117			10	14
OMM5121	31			6
OMM5126	21			
OMM5127	9		16	7

## Discussion

### Microsatellite marker development

Marker development strategies for the construction of high-density genetic maps typically utilize random or targeted approaches. Random approaches are commonly employed in the early phases of the map construction and are characterized by the use of sequence data not associated with mapping or functional annotation for marker development. In targeted approaches, commonly employed to increase marker density in a specific chromosome region or to map genes of interest, only sequence data meeting specified parameters with respect to mapping or function are utilized for marker development. Our approach for increasing the marker densities of rainbow trout genetic maps was a hybrid of random and targeted approaches. Although clones for marker development were not chosen based on functional annotation, the sequence data utilized were known to be transcribed. The benefit of this approach is that these microsatellites are Type I and II markers [5], serving to increase marker densities on both genetic and comparative maps. Similar strategies have been employed in the development of microsatellite markers for other agriculturally important animals including sheep, turkey, cattle, catfish, and pig [36-40].

### Cross amplification within the salmonidae

Salmonids are believed to have diverged from a common tetraploid ancestor some 25 million years ago [22]. As a result of this evolutionarily recent divergence, microsatellite markers can be used in the development of comparative genetic maps among the salmonidae. Cross-species amplification was obtained for 74 markers and ranged between 83% and 97% per species, with observed polymorphism that ranged between 36% and 82% per marker. Sampling additional individuals from multiple populations is likely to increase observations of polymorphism. This high level of cross-amplification and polymorphism should facilitate the development of comparative and genetic maps for the salmonids.

### Functional annotation

The RTGI was used to associate ESTs with functional annotation as their sequence data was previously included in RTGI Version 4.0. Unfortunately, 42% of the markers were not associated with any annotation, demonstrating an overall lack of functional annotation of the rainbow trout transcriptome.

### Genetic and comparative mapping

The goal of the activities outlined in this manuscript was to identify homologous regions of chromosomes between rainbow trout and species for which there is an abundance of genome information including whole genome sequence. Eight regions of homology were identified between trout and tetraodon, seven with human, and 10 with mouse (Table 2). Although mapping single loci does not identify segments of conserved synteny, the homologies reported in this paper are supported by the examination of direct comparative information between tetraodon and human and mouse. For instance, OMM5000 was observed to be homologous with TNI 8, HSA19, and MMU7. The NCBI human/mouse comparative map [41] reveals a homologous region between HSA 19 and MMU 7, and the tetraodon comparative map [42,43] reveals regions of homology between TNI8 and both HSA19 and MMU7. Similar analyses of comparative assignments in two or more species supported our findings for every marker reported.

## Conclusion

This project was initiated at a time where very little sequence data was publicly available for salmonid species. Now the RTGI contains over 150,000 ESTs which represent ~ 50,000 unique sequences. Current methods to develop new microsatellite markers from EST sequences would most likely replace hybridization with an *in silico *strategy on the RTGI data set. Therefore, the continuation of microsatellite marker development from expressed sequence tag data is feasible and will be useful for developing comparative maps with other salmonids and with better studied species.

## Methods

### Identification of cDNA clones with microsatellites

A rainbow trout normalized cDNA library was constructed using mRNA from brain, gill, liver, spleen, kidney, and muscle tissues. The library was plated, picked, and arrayed into 384-well plates. Sets of 72 plates were gridded onto single 20 cm^2 ^positively charged nylon membranes for hybridization experiments. One high-density membrane (representing 27,648 clones) was hybridized overnight at 65°C with radioactively (^32^P) labeled (GA)_11 _and (GT)_11 _oligonucleotide probes using standard protocols [44]. Membranes were removed from hybridization solution, washed, and exposed to storage phosphor screens for 1 hour. The phosphor screens were scanned on a Storm (Amersham Biosciences Corp, Piscataway, NJ) and positive clones identified.

### Sequencing and primer design

Positive clones were re-arrayed into 96-well plates and grown overnight. DNA was isolated for each clone using manufacturer's standard miniprep protocols for the BioRobot 8000 (QIAGEN, Valencia, CA, USA). Sequencing reactions were carried out using ABI Dye Terminator Chemistry (Applied Biosystems, Foster City, CA, USA) using SP6 and T7 primers. Sequencing reactions were purified and electrophoresed on an ABI3700. Sequences were trimmed for quality and vector using PHRED and Cross_match [32]. Consensus sequences were constructed for clones having multiple sequence data files. Those containing microsatellites were analyzed for redundancy within the dataset and previously discovered salmonid microsatellites using Vector NTI Suite 6.0 (InforMax, Bethesda, MD). PCR primer pairs were designed to amplify unique microsatellite sequences using Oligo 6.0 [45].

### PCR and genotyping

PCR primer pairs were obtained from commercial sources with the forward primers labeled with FAM, HEX, or NED. Primer pairs were optimized by varying annealing temperatures and MgCl_2 _concentrations to amplify in rainbow trout (Kamloop strain), the clone of origin, and a negative control with no DNA. Reactions (11 μl total volume) included 25 ng DNA, 1.5–2.5 mM MgCl_2_, 2.0 μM of each primer, 200 μM dNTPs, 1 × manufacturer's reaction buffer, and 0.5 unit AmpliTaq Gold Polymerase (ABI, Foster City, CA). Amplifications were conducted in an MJ Research PTC 200 DNA Engine thermal cycler (MJ Research, Waltham, MA) as follows: an initial denaturation at 94°C for 10 min, 36 cycles consisting of 94°C for 30 s, annealing temperature for 30 s, 72°C extension for 30 s; followed by a final extension of 72°C for 10 min. Successfully optimized primer pairs were used to amplify DNA from the three reference family parents [3] and five doubled haploid clonal lines (OSU, Arlee, Swanson, Hot Creek, and Clearwater [46]). Cross-species amplifications were attempted in two samples representing various other salmonids including cutthroat, Sockeye, Kokanee, Chinook, Atlantic salmon, brown trout, brook trout, and Artic char. PCR products were electrophoresed and verified by visualization in 3% agarose gels. PCR reactions were then combined according to label and size. Typical combinations of markers for capillary electrophoresis were made by combining PCR reactions for markers having alleles of at least 100 bp (based on agarose results) difference in size and different fluorescent labels. One microliter of each PCR product was added to 20 microliters of water, of which one microliter was added to 12 microliters of HiDi formamide and 0.5 microliters of ROX standard for genotyping for electrophoresis on an ABI PRISM3700 DNA Analyzer or an ABI PRISM 3100 Genetic Analyzer (Applied Biosystems, Foster City, CA, USA). Genescan output files were analyzed using Genotyper 3.5 software (Applied Biosystems, Foster City, CA, USA). Markers for which the parents of the reference families were informative were genotyped on the offspring. Markers not informative on the Sakamoto *et al*. [3] map having been associated with mapping annotation were genotyped on the reference families of Nichols *et al*. [2].

### Annotation

A FASTA file was generated containing clone sequence data for use in standalone BLAST with the goal of obtaining functional and mapping annotation. Functional annotation was associated by comparison to the RTGI Version 4.0 (Appendix 2) [31]. Mapping annotation was obtained by comparisons to sequence data from the Tetraodon Genome Browser [47] and zebrafish, fugu, human and mouse genome sequences from NCBI (Appendices 4 and 5) [48].

## Authors' contributions

CER conceived the study and participated in its design and coordination and drafted the manuscript. MFR, IC and YP assisted in genotyping analyses, RGD and KG conducted linkage analysis on the Sakamoto *et al*. [3] reference families and JD and RP conducted mapping in the doubled haploid crosses [2]. All authors read and approved the final manuscript.

## Supplementary Material

Additional File 1 Appendix 1. Microsatellite marker information including GenBank accessions, duplication status, allele size ranges, repeat motif, primer sequences, and optimized PCR conditions. Click here for file

Additional File 2 Appendix 2. Functional Annotation. Tentative annotation assigned to marker ESTs acquired via BLAST of the rainbow trout gene index version 4.0. Markers identified as homologues are annotated with gene or locus name symbols from UniProt [35] or NCBI. Click here for file

Additional File 3 Appendix 3. Mapping information for rainbow trout microsatellites. Each marker which was informative for mapping is included with cross, closest marker locus name, linkage group, and map position. Click here for file

Additional File 4 Appendix 4. *In silico *derived comparative mapping information I. BLAST was used to identify similar sequences between mouse, human, zebrafish, and pufferfish. Click here for file

Additional File 5 Appendix 5. *In silico *derived comparative mapping information II. BLAST was used to identify similar sequences with tetraodon. Click here for file
