# Supplementary material for: Comparative mapping of expressed sequence tags containing microsatellites in rainbow trout (Oncorhynchus mykiss)
Source: BMC Genomics. 2005 Apr 18;6:54. doi: 10.1186/1471-2164-6-54 (PMC1090573; doi:10.1186/1471-2164-6-54)
Supplement: Additional File 1 — Appendix 1. Microsatellite marker information including GenBank accessions, duplication status, allele size ranges, repeat motif, primer sequences, and optimized PCR conditions. [file 1471-2164-6-54-S1.doc]

## Appendix 1 - Microsatellite marker information

^1^Duplicated refers to the amplification of more than one amplicon in doubled haploid individuals.

^2^Number of alleles observed in 5 doubled haploid individuals and parents of mapping families.

^3^Allele size range in bp.

^4^A * indicates the repeat was imperfect in the clone of origin.

^5^Annealing Temperature in degrees Celsius.

| Locus | GenBank Accession | Dup^1^ | #  Alleles^2^ | Allele Size  Range^3^ | Repeat^4^ | Primer Sequences | AT^5^ | MgCl_2_ |
| --- | --- | --- | --- | --- | --- | --- | --- | --- |
| OMM5000 | CO805106 | Y | 5 | 239-280 | (CA)_18_ | F 5’aacagagcagtgaggggactgaga3’  R 5’caagtgatgttggtgcgaggg3’ | 58 | 2mM |
| OMM5001 | CO805107 | Y | 6 | 85-176 | (CA)_2_(GA)_29_* | F 5’gaagtctcagccagtggtaaagtc3’  R 5’ccctttacaagcctgatgtcat3’ | 58 | 2mM |
| OMM5002 | CO805108 | Y | 6 | 147-165 | (CA)_18_ | F 5’gggcttcctggaggactacttta3’  R 5’gccctgacagacagcaacatatag3’ | 58 | 2mM |
| OMM5003 | CO805109 | N | 6 | 162-179 | (GT)_11_ | F 5’tctaggctggtccttgtaggttgt3’  R 5’tcctggagaatgtcaagtccgt3’ | 58 | 2mM |
| OMM5004 | CO805110 | N | 3 | 192-202 | (GT)_11_ | F 5’agcttgtattaaagaggcttgaaa3’  R 5’caaattggaccgcacacta3’ | 58 | 2mM |
| OMM5005 | CO805111 | N | 4 | 193-209 | (AC)_14_ | F 5’tgaccagcacctccttatacctc3’c  R 5’ggcatcaaccaagagctaaaccaa3’ | 58 | 2mM |
| OMM5006 | CO805112 | N | 5 | 215-225 | (AG)_20_ | F 5’aaggcatcattggtgataacaagg3’  R 5’gggacgatgctttggctaaga3’ | 58 | 2mM |
| OMM5007 | CO805113 | N | 7 | 152-194 | (GT)_25_ | F 5’agatgcctgtcgagtgttg3’  R 5’gaggagcatcatttagagactaca3’ | 58 | 2mM |
| OMM5008 | CO805114 | N | 6 | 234-255 | (GT)_19_ | F 5’ctgtttcgttgtcctcatatcaacc3’  R 5’tccattatccaatcaggagagctctat3’ | 62 | 2mM |
| OMM5009 | CO805115 | Y | 8 | 295-355 | (GA)_44_ | F 5’caaggcctgaatccagctgtatta3’  R 5’tactttctctccctctccctcgtc3’ | 58 | 2mM |
| OMM5010 | CO805116 | N | 7 | 287-334 | (GT)_25_ | F 5’cccaacttgctgtccctaga3’  R 5’taacccacagaaagaaggtgttgt3’ | 62 | 2mM |
| OMM5011 | CO805117 | Y | 5 | 229-252 | (CA)_17_ | F 5’ggcacccagcaagtgatctacttc3’  R 5’ctgtgcgttcccagtggactct3’ | 62 | 2mM |
| OMM5012 | CO805118 | Y | 6 | 178-197 | (CA)_14_ | F 5’aggaccccacccacac3’  R 5’ttggagggtctatgctcg3’ | 62 | 2mM |
| OMM5013 | CA348663 | N | 5 | 92-112 | (GT)_17_ | F 5’agggtacaggaggtaaacag3’  R 5’aaactgatatggaggttgaac3’ | 62 | 2mM |
| OMM5014 | CO805119 | Y | 6 | 189-210 | (GT)_11_ | F 5’gggtctgaaaggagcatgg3’  R 5’ggaacctaacatgacgcaaca3’ | 62 | 2mM |
| OMM5017 | CO805122 | Y | 5 | 177-209 | (GT)_17_ | F 5’ttgagccaaacatgcctc3’  R 5’cacagcatctagacagttccc3’ | 58 | 2mM |
| OMM5018 | CO805123 | N | 4 | 191-197 | (TA)_3_(CA)_19_* | F 5’gaaggaacggaacagagtggtaatcac3’  R 5’tcggacaggtaactggaacggat3’ | 58 | 2mM |
| OMM5019 | CO805124 | Y | 5 | 254-275 | (GT)_11_ | F 5’catgctgcctctcaccgttta3’  R 5’aacacacccagcatccaacc3’ | 58 | 2mM |
| OMM5020 | CO805125 | N | 2 | 261-263 | (AC)_13_ | F 5’agccagccggtctgtttagtc3’  R 5’tggccccataacacaggagtatta3’ | 58 | 2mM |
| OMM5023 | CA348610 | Y | 3 | 97-128 | (CT)_8_(CA)_29_* | F 5’attaggcaaccctcatgaactgta3’  R 5’tgccattgagtgtctttaagcac3’ | 62 | 2mM |
| OMM5024 | CA348611 | N | 6 | 210-239 | (GT)_16_ | F 5’caccgtcaccatcttttctccctatg3’  R 5’gtcagtgtccctcactacttgccaatc3’ | 62 | 2mM |
| OMM5025 | CA349137 | Y | 4 | 160-182 | (CA)_19_ | F 5’ggaggacagttaaaggcatcgcgcttc3’  R 5’cgtcatatcttcaagctccgcgagagg3’ | 54 | 2mM |
| OMM5026 | CA349138 | N | 5 | 216-323 | (CA)_11_ | F 5’tgactcaagtctttgcctactcac3’  R 5’ggtcaggatgtggcagtaatg3’ | 56 | 2mM |
| OMM5028 | CA349149 | Y | 6 | 284-306 | (GT)_13_ | F 5’cagcaccatctgtaccggtcatc3’  R 5’caggttatccgagtggttctaatgtcc3’ | 62 | 1.5mM |
| OMM5029 | CA348622 | Y | 5 | 208-242 | (CA)_24_ | F 5’tgctccctctggactatctagcct3’  R 5’gacgcacacagacatatacatgcc3’ | 62 | 1.5mM |
| OMM5030 | CA349141 |  | 4 | 149-165 | (CA)_15_ | F 5’gggtgctctacacacatacacaat3’  R 5’tggaggtatcttgtggtcagtct3’ | 58 | 2mM |
| OMM5031 | CA349143 | Y | 4 | 129-144 | (CA)_10_ | F 5’gaaacctccccatatcattg3’  R 5’cggcagaatatctccataagt3’ | 58 | 2mM |
| OMM5032 | CA349143 | N | 5 | 174-189 | (CA)_13_ | F 5’gccttccaatgatcctagag3’  R 5’ccacactatgtgaccctcact3’ | 62 | 2mM |
| OMM5033 | CA349148 | N | 7 | 225-284 | (CA)_28_ | F 5’tggtcagatatacagctccaacctc3’  R 5’acatgtaagccaatcaacacatagact3’ | 58 | 1.5mM |
| OMM5034 | CA349160 | Y | 5 | 247-276 | (GA)_9_ | F 5’acaaagctgccatcagataacatc3’  R 5’gtacggttgaattgagccagtcta3’ | 58 | 2mM |
| OMM5035 | CA348633 | N | 4 | 188-211 | (GT)_17_ | F 5’tacctcttgaactcgttaacc3’  R 5’gcacggctcatacaca3’ | 58 | 2mM |
| OMM5036 | CA349152 | N | 6 | 266-291 | (AC)_13_ | F 5’cgaaaacacacaagatggaggcggag3’  R 5’agacctcatttctgatcctgccgctgt3’ | 56 | 1.5mM |
| OMM5037 | CA348625 | Y | 7 | 257-274 | (CA)_15_ | F 5’accgaggtagaggaagaagcttta3’  R 5’ctcatagacaaagccttgaacgag3’ | 58 | 2mM |
| OMM5039 | CA349159 | N | 4 | 262-285 | (GT)_11_ | F 5’acagaaatggttcaggtccttcatagt3’  R 5’cagcagcaagagttcttcagaacac3’ | 58 | 2mM |
| OMM5041 | CA348635 | Y | 4 | 133-196 | (CT)_8_(GT)_11_(GA)_4_* | F 5’ctggggactcttggaact3’  R 5’agcgtaaagcgtcatgg3’ | 58 | 2mM |
| OMM5042 | CA349165 | N | 5 | 130-161 | (AC)_13_ | F 5’ggcccactttttatgacatgatt 3’  R 5’tcctataagcccaacatggtacag3’ | 58 | 2mM |
| OMM5043 | CA349167 | N | 5 | 114-123 | (CA)_11_ | F 5’aactgtttttgcctccaccctat3’  R 5’gctccctcctttgtttatttttaggtt3’ | 58 | 2mM |
| OMM5044 | CA349174 | N | 6 | 234-269 | (CA)_27_ | F 5’gcccacataaacacagacgca 3’  R 5’gtccggtggttgagcagtaca3’ | 58 | 2mM |
| OMM5045 | CA348654 |  | 5 | 263-302 | (GT)_11_ | F 5’ccaatgcaactgaccgttc3’  R 5’atctcatcacaatggaccagc3’ | 54 | 2mM |
| OMM5046 | CA349184 |  | 6 | 258-277 | (TA)_30_ | F 5’gcttacggccttgcctacaccttgc3’  R 5’tgtagttgacagtgagggggcgttttt3’ | 58 | 2mM |
| OMM5047 | CA349175 | N | 6 | 259-278 | (GT)_12_ | F 5’actttcagcagcatctggtca3’  R 5’cctggtcctcagcgttcat3’ | 58 | 2mM |
| OMM5050 | CA348659 | N | 6 | 245-275 | (GT)_13_ | F 5’acaactggaataggaacgcaaagc3’  R 5’caattctctcatcctcgccactc3’ | 52 | 1.5mM |
| OMM5051 | CA349193 | Y | 7 | 178-207 | (CA)_10_ | F 5’cctccaccctataccaccccataa3’  R 5’gggggaaaccattcgagatgtt3’ | 52 | 1.5mM |
| OMM5053 | CA349198 |  | 2 | 247 | (GT)_21_ | F 5’tctatggacaaactgggagtaaatgcc3’  R 5’gccctctcattgtgtttctgtatagcc3’ | 56 | 1.5mM |
| OMM5054 | CA348677 | Y | 3 | 121-262 | (CT)_27_ | F 5’gtttctatttccaccctcaccgctgat3’  R 5’gggcaaaatttcttgtcaagccaacc3’ | 48 | 2mM |
| OMM5055 | CA348686 | N | 5 | 224-232 | (AC)_15_ | F 5’ggaacagggctgcattagctttg3’  R 5’cgccataggtcttgttcaggtaca3’ | 52 | 1.5mM |
| OMM5056 | CA349207 | N | 4 | 198-215 | (GT)_13_ | F 5’tcaccatcaccttcatcgcct3’  R 5’acatgctgccctttgacggag3’ | 58 | 1.5mM |
| OMM5057 | CA348687 | Y | 4 | 198-213 | (GT)_40_ | F 5’tgtgtttgttttagtgtctgtccg3’  R 5’ccatttgagatacagacgctgtg3’ | 54 | 2mM |
| OMM5058 | CA348781 | N | 3 | 196-213 | (CA)_11_ | F 5’ cacccatcagatttgtaagagcgt3’  R 5’tcctatgcgtttgctttcgtg3’ | 48 | 2mM |
| OMM5059 | CA348697 | N | 5 | 141-167 | (GT)_32_ | F 5’ccgcagcttccgttctactcc3’  R 5’tgactacccatgatgcacggc3’ | 48 | 1.5mM |
| OMM5060 | CA348688 | Y | 5 | 180-214 | (AC)_18_* | F 5’tctcgggccaaaccttcttattgc3’  R 5’agccactacatctccacgccctt3’ | 48 | 1.5mM |
| OMM5061 | CA348691 | N | 4 | 275-281 | (CA)_12_ | F 5’gcgttgggagagaacaatacc3’  R 5’cccatcacaccagttgcc3’ | 52 | 2mM |
| OMM5062 | CA348777 | Y | 7 | 224-251 | (CA)_12_ | F 5’tgcagtaacctgaaggtccaatggg3’  R 5’aaggaggagggagagagctacggagac3’ | 52 | 2mM |
| OMM5063 | CA348783 | N | 5 | 163-210 | (GT)_15_ | F 5’ggggtgatgatggaaagattagtg3’  R 5’gggaaaaataactgatggacggt3’ | 48 | 2mM |
| OMM5064 | CA348707 | N | 3 | 288-297 | (GT)_12_ | F 5’tgtctgaagaacctgcctatctgt3’  R 5’gctggtgctgaccttggtagtg3’ | 52 | 2mM |
| OMM5065 | CA348787 | N | 5 | 151-211 | (CA)_56_ | F 5’gcgcaaacaatgtaactaacactc3’  R 5’gtatatcaccgacaattcagcga3’ | 48 | 2mM |
| OMM5067 | CA348790 | Y | 6 | 153-195 | (CA)_13_ | F 5’cataagcagaaatcaggggtaaca3’  R 5’gggcataagctatgtaatttacgc3’ | 48 | 2mM |
| OMM5069 | CA348796 | Y | 4 | 248-298 | (GT)_11_ | F 5’gaaccacatgggaaatgttgaggttag3’  R 5’ctctccctcagtgatggacttaaacg3’ | 52 | 2mM |
| OMM5072 | CA348719 | Y | 6 | 151-195 | (CA)_13_ | F 5’agacagccaaccactgatacc3’  R 5’agaaacatggcaaacgatga3’ | 52 | 2mM |
| OMM5074 | CA348721 | Y | 3 | 176-246 | (GT)_14_ | F 5’tcgctttgggtagaagttgcctttaac3’  R 5’aacattaagaacgagtggaatcacgc3’ | 52 | 2mM |
| OMM5075 | CA348807 | N | 4 | 195-209 | (GT)_12_ | F 5’agattcaccacgtatcacggaatg3’  R 5’tcttcacatcctactcaatgatcgacc3’ | 52 | 2mM |
| OMM5077 | CA348730 | N | 3 | 377-381 | (CA)_11_ | F 5’tatgcgctagactacaacgag3’  R 5’tctgaggggcaatgtaagt3’ | 52 | 2mM |
| OMM5087 | CA348752 |  | 3 | 257-284 | (GT)_10_ | F 5’ggactgctggggttgattga3’  R 5’acacacatctcaggcctcctaagt3’ | 48 | 2mM |
| OMM5088 | CA348759 | N | 6 | 166-181 | (GT)_19_ | F 5’atctctcgtctttcctgtcttcgt3’  R 5’actctggctgtgcattgtgg3’ | 58 | 2mM |
| OMM5090 | CA348849 | N | 3 | 260-269 | (GT)_20_(CA)_5_* | F 5’aacaaggcaagcaacaagcataac3’  R 5’gggagcaggtatctttcggtct3’ | 48 | 1.5mM |
| OMM5091 | CA348850 | Y | 5 | 277-319 | (GA)_49_(GT)_11_* | F 5’gcaggaaaaacacccagatacaa3’  R 5’acactggctggtgtcgttacatta3’ | 58 | 2mM |
| OMM5092 | CA348764 | N | 3 | 172-195 | (GT)_10_ | F 5’cgccacaacagcagcacttaga3’  R 5’caaaggccagcgtttagatagcac3’ | 58 | 2mM |
| OMM5093 | CA348861 | Y | 4 | 274-292 | (GT)_12_ | F 5’ttaatcacacagcattagtagtcagcc3’  R 5’gagttagatgacctggttaatgcc3’ | 48 | 1.5mM |
| OMM5095 | CA348914 | N | 3 | 89-144 | (GT)_32_* | F 5’gggatgttcaagtagcatga3’  R 5’atctgtacacaatggcaacac3’ | 58 | 2mM |
| OMM5097 | CA348868 |  | 3 | 284-292 | (AC)_12_ | F 5’tcatcatcataaacgggtcac3’  R 5’tgccgacgttactcataactg3’ | 58 | 2mM |
| OMM5098 | CA348879 | N | 5 | 116-133 | (GT)_22_ | F 5’cgcatcagaatgaccaggatacta3’  R 5’cacactgatagcacacacgcatac3’ | 48 | 1.5mM |
| OMM5099 | CA348959 | Y | 4 | 213-281 | (CT)_24_ | F 5’gaatcgaccaacaagaccatc3’  R 5’ttggcacagaaaagacgtaca3’ | 48 | 2mM |
| OMM5100 | CA348952 | N | 4 | 169-181 | (CA)_12_ | F 5’tgctggatactggagctactt3’  R 5’tctggtttcagggactgc3’ | 48 | 2mM |
| OMM5102 | CA348955 | Y | 4 | 296-304 | (AC)_11_ | F 5’attccaaataacaggtgctactggtc3’  R 5’ctggttaactaggcaactgattgtgtc3’ | 48 | 2mM |
| OMM5104 | CA348965 | Y | 4 | 136-169 | (GT)_10_ | F 5’ccagattggcttgtgttgtataac3’  R 5’ataaggaactccctagttgaccac3’ | 48 | 2mM |
| OMM5106 | CA348902 | Y | 6 | 257-284 | (CA)_16_ | F 5’ggtatgatgcctctgaatgaacagtat3’  R 5’accagttggtgtttaactcatatcagc3’ | 52 | 2mM |
| OMM5107 | CA348985 | N | 3 | 254-268 | (GT)_11_ | F 5’agagcaacaaatccacgtgag3’  R 5’atcagccataactgccatactgta3’ | 52 | 2mM |
| OMM5108 | CA349062 | N | 4 | 266-284 | (GT)_12_ | F 5’acactatcccatagttgactgacg3’  R 5’ctggagaaatgtggcacaataa3’ | 48 | 2mM |
| OMM5109 | CA348993 | N | 5 | 262-274 | (AC)_10_ | F 5’gtttcacaaagtcataacgagcag3’  R 5’gttggcacaaggcattatacc3’ | 48 | 2mM |
| OMM5112 | CA349005 | N | 4 | 198-206 | (GT)_10_ | F 5’cccttcccacaatcctgacctta3’  R 5’catgctgatcctgctggtactcct3’ | 48 | 2mM |
| OMM5113 | CA349018 | N | 5 | 249-299 | (CA)_33_(CT)_15_* | F 5’tcggtaacaagtcctctagaccaca3’  R 5’cagagacctagactgagtcatgtcctg3’ | 52 | 2mM |
| OMM5116 | CA349104 | Y | 4 | 272-280 | (GT)_12_ | F 5’aacatgtccagccctggtctgtta3’  R 5’tggaaggggagacaaaccgtta3’ | 48 | 2mM |
| OMM5117 | CA349111 | N | 2 | 150-152 | (GT)_13_ | F 5’gcgagtgcaagaggaaacaagtac3’  R 5’cgcagagccattgagagatactgt3’ | 48 | 2mM |
| OMM5121 | CA349040 | Y | 2 | 198-221 | (AG)_15_ | F 5’cttcaggctttattgtgtttgacatgc3’  R 5’gccaacatgtaacaccactagctgc3’ | 58 | 2mM |
| OMM5122 | CO805126 | N | 2 | 361-377 | (TA)_6_(CA)_6_* | F 5’cttgcttcgtgtttttcttgccac3’  R 5’aggcctgcctctcaaatgtagtgtact3’ | 52 | 2mM |
| OMM5124 | CA349048 | N | 5 | 258-281 | (GT)_10_ | F 5’tctctttgtaacgtagtgctgcgagtg3’  R 5’tgtggaggtgactgtcgggtatct3’ | 52 | 2mM |
| OMM5125 | CO805127 | N | 2 | 247-257 | (CA)_13_ | F 5’aaagccctcattgtgataacactg3’  R 5’gcgctgtgtagaacggaatc3’ | 52 | 2mM |
| OMM5126 | CO805128 | N | 6 | 281-295 | (GT)_10_ | F 5’cacactatttgggacgcaca3’  R 5’cccaacagttgagtctccatagtt3’ | 52 | 2mM |
| OMM5127 | CO805129 | N | 5 | 204-217 | (GT)_11_ | F 5’tgacatgggcgactgtactgt3’  R 5’ctcaagcattccggttagtgg3’ | 48 | 1.5mM |
