# Supplementary material for: Comparative mapping of expressed sequence tags containing microsatellites in rainbow trout (Oncorhynchus mykiss)
Source: BMC Genomics. 2005 Apr 18;6:54. doi: 10.1186/1471-2164-6-54 (PMC1090573; doi:10.1186/1471-2164-6-54)
Supplement: Additional File 2 — Appendix 2. Functional Annotation. Tentative annotation assigned to marker ESTs acquired via BLAST of the rainbow trout gene index version 4.0. Markers identified as homologues are annotated with gene or locus name symbols from UniProt [35] or NCBI. [file 1471-2164-6-54-S2.doc]

## Appendix 2 - Functional annotation.

Tentative annotation assigned to marker ESTs acquired via BLAST of the rainbow trout gene index version 4.0. Markers identified as homologues are annotated with gene or locus name symbols from UniProt [34] or NCBI. Hit Definitions, expectation values (E-Value), number of identical base pairs (Identity), and alignment lengths (Align Length) are given.

| **Locus** | **Hit Definition** | **E-Value** | **Identity** | **Align**  **Length** |
| --- | --- | --- | --- | --- |
| OMM5000 | TC62497 similar to UP|AAH64215 (AAH64215) MGC76117 protein, partial (77%) | 0 | 495 | 496 |
| OMM5001 | TC48447 | 6.4E-117 | 218 | 219 |
| OMM5002  (yrk) | TC47580 homologue to UP|Q8AWF1 (Q8AWF1) Yes-relayed kinase, partial (51%) | 0 | 608 | 609 |
| OMM5003 | TC62779 | 0 | 317 | 320 |
| OMM5004 | TC56823 | 0 | 391 | 392 |
| OMM5005  (Zgc:66415) | TC55641 homologue to UP|Q7SX89 (Q7SX89) CLLL6 protein, complete | 0 | 623 | 623 |
| OMM5006 | TC58658 weakly similar to UP|AAH65890 (AAH65890) Zgc:77906 protein, partial (14%) | 1.1E-138 | 248 | 248 |
| OMM5007 | TC51208 | 0 | 356 | 356 |
| OMM5008 | TC67172 UP|Q7PI42 (Q7PI42) ENSANGP00000024316 (Fragment), partial (7%) | 0 | 476 | 477 |
| OMM5009 | TC53122 | 6.1E-123 | 246 | 246 |
| OMM5010 | TC64693 similar to SP|Q9UQ16|DYN3_HUMAN Dynamin 3 (Dynamin, testicular) (T-dynamin). {Homo sapiens;} , partial (4%) | 0 | 515 | 518 |
| OMM5011 | TC60799 similar to UP|Q8NH31 (Q8NH31) Seven transmembrane helix receptor, partial (6%) | 0 | 351 | 353 |
| OMM5012 | TC48555 | 2.88E-76 | 179 | 179 |
| OMM5013 | TC50062 similar to UP|Q7PI42 (Q7PI42) ENSANGP00000024316 (Fragment), partial (8%) | 5E-142 | 263 | 266 |
| OMM5014 | TC56364 UP|Q9UPD4 (Q9UPD4) Ndr Ser/Thr kinase-like protein (Fragment), partial (8%) | 0 | 575 | 577 |
| OMM5015 | TC57343 | 0 | 458 | 459 |
| OMM5016 | TC51061 similar to GB|AAH01584.1|16306780|BC001584 LOC124245 protein {Homo sapiens;} , partial (4%) | 2.7E-126 | 238 | 245 |
| OMM5017 | TC49320 weakly similar to SP|P07306|LECH_HUMAN Asialoglycoprotein receptor 1 (Hepatic lectin H1) (ASGPR) (ASGP-R). {Homo sapiens;} , partial (7%) | 0 | 355 | 358 |
| OMM5018 | TC58410 similar to GB|AAH04681.1|13435609|BC004681 Lrpprc protein {Mus musculus;} , partial (34%) | 1.6E-128 | 231 | 232 |
| OMM5019 | TC65376 similar to GB|AAH44386.2|28277526|BC044386 nudE nuclear distribution gene E homolog like 1 (A. nidulans) B {Danio rerio;} , partial (25%) | 0 | 431 | 431 |
| OMM5020 | TC51867 similar to GB|AAC51216.1|1934601|HSU91616 I kappa B epsilon {Homo sapiens;} , partial (4%) | 0 | 458 | 460 |
| OMM5021 | TC66103 | 8.47E-66 | 143 | 152 |
| OMM5023 | CA348610 | 1.1E-93 | 173 | 173 |
| OMM5024 | TC63286 | 0 | 509 | 511 |
| OMM5025 | CA349137 | 2.3E-153 | 337 | 338 |
| OMM5026 | CA349138 | 0 | 340 | 342 |
| OMM5028 | TC55595 similar to UP|Q8NH31 (Q8NH31) Seven transmembrane helix receptor, partial (7%) | 0 | 406 | 409 |
| OMM5029 | TC48232 UP|AAQ96223 (AAQ96223) LRRGT00010, partial (17%) | 7.7E-178 | 314 | 314 |
| OMM5030 | CA349141 | 0 | 527 | 527 |
| OMM5031 | TC64300 similar to UP|BAC97813 (BAC97813) Slingshot-2L, partial (3%) | 0 | 357 | 361 |
| OMM5032 | TC64300 similar to UP|BAC97813 (BAC97813) Slingshot-2L, partial (3%) | 0 | 357 | 361 |
| OMM5033 | TC56770 | 0 | 374 | 379 |
| OMM5034 | TC66565 | 8.8E-153 | 272 | 272 |
| OMM5035 | TC52738 | 0 | 566 | 567 |
| OMM5036  (TREML2) | TC56588 homologue to UP|Q8IWY0 (Q8IWY0) TREM-like transcript 2, partial (5%) | 0 | 404 | 406 |
| OMM5037  (AAR92146) | TC65289 homologue to UP|AAR92146 (AAR92146) Tumor differentiation factor, partial (11%) | 0 | 437 | 441 |
| OMM5038 | TC55229 similar to AAH65846 (AAH65846) LIM domain binding 3, partial (90%) | 0 | 624 | 630 |
| OMM5039 | TC53695 similar to UP|Q7PFX3 (Q7PFX3) ENSANGP00000024497 (Fragment), partial (5%) | 5E-108 | 197 | 197 |
| OMM5041  (Pdcd10) | TC48172 homologue to UP|Q8VE70 (Q8VE70) Programmed cell death 10, complete | 0 | 551 | 553 |
| OMM5042 | TC66946 | 0 | 399 | 410 |
| OMM5043 | TC62514 similar to UP|AAR20772 (AAR20772) At5g14440, partial (5%) | 4.2E-167 | 367 | 370 |
| OMM5044 | TC64952 UP|Q69566 (Q69566) U88, partial (6%) | 1.3E-145 | 270 | 272 |
| OMM5045 | TC53100 similar to GB|AAH46374.1|28385985|BC046374 dnm1l-prov protein {Xenopus laevis;} , partial (18%) | 0 | 435 | 436 |
| OMM5046 | CA349184 | 1.3E-151 | 369 | 369 |
| OMM5047 | TC53679 UP|Q64150 (Q64150) Nuclear localization signal binding protein, partial (7%) | 0 | 444 | 447 |
| OMM5050 | CA348659 | 0 | 473 | 474 |
| OMM5051 | TC62515 | 1.2E-139 | 289 | 295 |
| OMM5053 | TC63408 weakly similar to SP|Q62234|MYM1_MOUSE Myomesin 1 (Skelemin). {Mus musculus;}, partial (9%) | 6.6E-166 | 294 | 294 |
| OMM5054 | TC60223 weakly similar to UP|Q8RWN3 (Q8RWN3) Protein kinase-like protein, partial (4%) | 1.92E-70 | 134 | 134 |
| OMM5055 | TC61504 UP|Q64150 (Q64150) Nuclear localization signal binding protein, partial (8%) | 0 | 505 | 507 |
| OMM5056 | TC56464 weakly similar to UP|AAH65451 (AAH65451) Zgc:77785 protein, partial (44%) | 0 | 390 | 395 |
| OMM5057 | TC52959 weakly similar to UP|Q69566 (Q69566) U88, partial (11%) | 1E-127 | 230 | 230 |
| OMM5058 | TC66689 similar to UP|AAN61065 (AAN61065) Doublesex-and mab-3-related transcription factor 1 b2, partial (5%) | 0 | 425 | 425 |
| OMM5059 | TC56777 weakly similar to SP|P20291|FLAP_RAT 5-lipoxygenase activating protein (FLAP) (MK-886-binding protein). {Rattus norvegicus;}, partial (87%) | 0 | 408 | 408 |
| OMM5060 | TC65543 UP|Q7PG24 (Q7PG24) ENSANGP00000023115, partial (14%) | 0 | 427 | 430 |
| OMM5061 | TC67876 UP|Q61402 (Q61402) Gcap1 protein (Fragment), partial (12%) | 0 | 380 | 381 |
| OMM5062 | TC48119 similar to UP|AAH65338 (AAH65338) Zgc:77312 protein, partial (69%) | 0 | 378 | 380 |
| OMM5063 | CA348783 | 0 | 374 | 374 |
| OMM5064 | TC53678 similar to UP|AAH65326 (AAH65326) Zgc:77228 protein, partial (4%) | 0 | 408 | 410 |
| OMM5065 | CA348787 | 2.5E-128 | 231 | 231 |
| OMM5067 | TC59894 similar to UP|Q9DG20 (Q9DG20) POP1D protein, partial (11%) | 0 | 464 | 468 |
| OMM5069 | CA348796 | 0 | 501 | 501 |
| OMM5072 | TC56095 weakly similar to UP|AAH61672 (AAH61672) MGC68826 protein, partial (21%) | 2.8E-131 | 245 | 248 |
| OMM5074 | TC59075 UP|AAR31136 (AAR31136) LD03248p, partial (12%) | 0 | 351 | 351 |
| OMM5075 | CA348807 | 0 | 349 | 350 |
| OMM5077  (HPRT) | TC55247 homologue to SP|Q9W719|HPRT_CHICK Hypoxanthine-guanine phosphoribosyltransferase (HGPRT)(HGPRTase). {Gallus gallus;} , complete | 0 | 426 | 431 |
| OMM5087 | TC62509 weakly similar to GB|AAL56659.1|18032249|AF231130 NUP196 nucleoporin {Homo sapiens;} , partial (10%) | 0 | 495 | 501 |
| OMM5088  (MUSIGHV01B) | TC53955 homologue to GB|AAA38496.1|554015|MUSIGHV01B Ig heavy chain precursor {Mus musculus;} , partial (28%) | 0 | 378 | 378 |
| OMM5089 | TC63583 | 1.17E-65 | 129 | 130 |
| OMM5090 | CA348849 | 3.67E-84 | 208 | 208 |
| OMM5091 | CA348850 | 1.49E-83 | 207 | 207 |
| OMM5092 | TC47372 similar to UP|Q801E8 (Q801E8) Cytosolic malate dehydrogenase A, complete | 8.87E-76 | 143 | 143 |
| OMM5093 | TC64145 similar to GB|AAH10087.1|14603253|BC010087 phosphoglucomutase 2 {Homo sapiens;} , partial (72%) | 0 | 434 | 443 |
| OMM5095 | TC48937 | 2.8E-168 | 298 | 298 |
| OMM5097 | TC57635 | 1.1E-173 | 307 | 307 |
| OMM5098 | TC48232 UP|AAQ96223 (AAQ96223) LRRGT00010, partial (17%) | 0 | 474 | 478 |
| OMM5099  (FAM49B) | TC64647 homologue to GB|AAH16345.1|16740986|BC016345 BM-009 protein {Homo sapiens;} , partial (33%) | 0 | 386 | 386 |
| OMM5100 | TC68802 | 0 | 615 | 616 |
| OMM5102 | TC48446 | 2.8E-131 | 236 | 236 |
| OMM5104 | TC57318 | 0 | 391 | 392 |
| OMM5106 | CA348902 | 6.7E-135 | 242 | 242 |
| OMM5107 | TC62941 | 0 | 373 | 374 |
| OMM5108 | TC57122 | 2.2E-150 | 268 | 268 |
| OMM5109 | TC67467 | 0 | 344 | 348 |
| OMM5112 | CA349005 | 0 | 411 | 411 |
| OMM5113 | TC47588 similar to GB|AAH31846.1|21594729|BC031846 SEC63-like {Mus musculus;}, partial (19%) | 9.3E-122 | 223 | 224 |
| OMM5116 | TC66250 similar to UP|Q7PG24 (Q7PG24) ENSANGP00000023115, partial (19%) | 4.4E-133 | 239 | 239 |
| OMM5117 | TC55229 similar to AAH65846 (AAH65846) LIM domain binding 3, partial (90%) | 4.1E-164 | 400 | 435 |
| OMM5121 | TC62667 similar to UP|Q92046 (Q92046) PREPROTRYPSIN precursor (Alpha- and beta-trypsin) , complete | 0 | 363 | 372 |
| OMM5122 | TC54245 | 1.34E-40 | 106 | 112 |
| OMM5124 | CA349048 | 0 | 322 | 322 |
| OMM5125 | TC56382 | 0 | 584 | 591 |
| OMM5126 | TC57197 | 0 | 605 | 609 |
| OMM5127 | TC63737 similar to UP|AAH63949 (AAH63949) Major vault protein, partial (42%) | 0 | 494 | 495 |
