# Supplementary material for: Comparative mapping of expressed sequence tags containing microsatellites in rainbow trout (Oncorhynchus mykiss)
Source: BMC Genomics. 2005 Apr 18;6:54. doi: 10.1186/1471-2164-6-54 (PMC1090573; doi:10.1186/1471-2164-6-54)
Supplement: Additional File 3 — Appendix 3. Mapping information for rainbow trout microsatellites. Each marker which was informative for mapping is included with cross, closest marker locus name, linkage group, and map position. [file 1471-2164-6-54-S3.doc]

Appendix 3. Mapping information for rainbow trout microsatellites. Each marker which was informative for mapping is included with cross, closest marker locus name, linkage group, and map position.

| **Locus** | **Cross** | **Closest Marker** | **Linkage Group** | **Distance**  **(cM)** |
| --- | --- | --- | --- | --- |
| OMM5000 | OSU x Clearwater | ACGATC351o | 27 | 0 |
| OMM5003 | OSU x Clearwater | AGCATC160ac | 23 | 3.0 |
| OMM5005 | OSU x Clearwater | ACCAAG9 | 11 | 1.2 |
| OMM5012 | 44m | OmyRGT18TUF | 23 | 23.7 |
| OMM5017 | OSU x Arlee | Pataatc225o | 20 | 15.7 |
| OMM5019 | OSU x Clearwater | Agcagc2 | 9 | 3.8 |
| OMM5023 | 44f | AGG/CTA109 | 22 | 26.1 |
|  | 44m | OMM5107 | 22 | 35.6 |
| OMM5025 | 44m | Ogo8UW | 8 | 49.6 |
| OMM5026 | 44m | OMM1201 | 29 | 88.3 |
| OMM5029 | 25f | OMM1277 | 12+2 | 42 |
|  | 25m | AGG/CAC194 | 12 | 19.5 |
| OMM5033 | 25f | AAG/CTG125 | 16 | 28 |
|  | 25m | Omy77DU | 16 | 23.4 |
|  | 44f | ACG/CTC356 | una21 | 0 |
|  | 44m | OMM1290 | 16 | 0 |
| OMM5034 | 44f | OMM1223 | 19+3 | 68.8 |
| OMM5041 | 44m | Omi173TUF | 12 | 45.5 |
| OMM5045 | OSU x Arlee | Etaccac117a | 19 | 3.0 |
| OMM5057 | 25f | OMM5127 | 9 | 0 |
|  | 25m | OMM1267 | 9 | 3.8 |
|  | 44f | AAC/CTT127 | 9 | 91.8 |
|  | 44m | OMM1368 | 9 | 9.3 |
| OMM5062 | 25f | OMM1108 | 27 | 8 |
|  | 25m | AAC/CAC348 | 27 | 19.5 |
|  | 44f | Omy1INRA | una20 | 0 |
| OMM5065 | 44f | AAC/CTC104 | 25 | 60.9 |
|  | 44m | OmyRGT7TUF | 25 | 4.3 |
| OMM5077 | OSU x Arlee |  | 25 |  |
| OMM5088 | 25f | OMM1223 | 19 | 58 |
|  | 25m | OMM1223 | 19 | 13.5 |
| OMM5090 | 44m | OmyFGT2TUF | 21 | 36.5 |
| OMM5099 | 44f | OmyRGT17TUF | 7 | 101.1 |
| OMM5100 | 44f | OMM1175 | 15 | 14.1 |
|  | 44m | BHMS339 | 15 | 44.4 |
| OMM5106 | 25m | OMM1134/i | 14 | 17.5 |
|  | 44m | OmyRGT43TUF | 14 | 1 |
| OMM5107 | 44m | OmyOGT4TUF | 22 | 32.5 |
| OMM5108 | 44f | BHMS205/i | una1 | 0 |
| OMM5109 | 25m | OmyRGT1TUF | 31 | 13.5 |
| OMM5112 | 25f | ACA/CTA125 | 23 | 66 |
|  | 25m | AGC/CTC62 | 23 | 44.9 |
|  | 44m | OmyFGT23TUF | 23 | 63.8 |
| OMM5121 | 44m | OmyFGT8/iiTUF | 31 | 19.4 |
| OMM5126 | 44f | AAG/CTA68 | 21 | 3.1 |
|  | 44m | Omi186TUF | 21 | 50.4 |
| OMM5127 | 25f | GH1(INRA) | 9 | 36 |
|  | 25m | Ogo7/iUW | 9 | 5.7 |
