# Supplementary material for: Comparative mapping of expressed sequence tags containing microsatellites in rainbow trout (Oncorhynchus mykiss)
Source: BMC Genomics. 2005 Apr 18;6:54. doi: 10.1186/1471-2164-6-54 (PMC1090573; doi:10.1186/1471-2164-6-54)
Supplement: Additional File 4 — Appendix 4. In silico derived comparative mapping information I. BLAST was used to identify similar sequences between mouse, human, zebrafish, and pufferfish. [file 1471-2164-6-54-S4.doc]

Appendix 4. *In silico* derived comparative mapping information using BLAST to identify similar sequences between mouse, human, zebrafish, and pufferfish.

| Locus | Organism | DB^a^ | Translation/Gene/contig/scaffold | Basepair | Status | chr^b^ | Score | E - Value^c^ | Identities | Positives |
| --- | --- | --- | --- | --- | --- | --- | --- | --- | --- | --- |
| OMM5000 | Human | pep | ENSP00000253237 | 53640874 | known | HSA19 | 609 | 9.8E-60 | 115/169 (68%) | 132/169 (78%) |
|  |  |  | ENSG00000105447 |  |  |  |  |  |  |  |
|  |  |  | AC008403.8.1.190076 |  |  |  |  |  |  |  |
|  |  |  |  |  |  |  |  |  |  |  |
|  | Mouse | pep | ENSMUSP00000038526 | 34467771 | known | MMU7 | 606 | 2.4E-59 | 115/169 (68%) | 131/169 (77%) |
|  |  |  | ENSMUSG00000040259 |  |  |  |  |  |  |  |
|  |  |  | CAAA01220486.1.1.6668 |  |  |  |  |  |  |  |
|  |  |  |  |  |  |  |  |  |  |  |
|  | Pufferfish | pep | SINFRUP00000129211 | 16572 | known |  | 806 | 1.7E-80 | 150/164 (91%) | 157/164 (95%) |
|  |  |  | SINFRUG00000122358 |  |  |  |  |  |  |  |
|  |  |  | scaffold_1961 |  |  |  |  |  |  |  |
|  |  |  |  |  |  |  |  |  |  |  |
|  | Zebrafish | pep | ENSDARP00000023040 | 161476 | novel |  | 566 | 3.4E-55 | 111/165 (67%) | 133/165 (80%) |
|  |  |  | ENSDARG00000004806 |  |  |  |  |  |  |  |
|  |  |  | ctg9554.1 |  |  |  |  |  |  |  |
|  |  |  |  |  |  |  |  |  |  |  |
| OMM5002 | Human | pep | ENSP00000012102 | 112028512 | known | HSA6 | 789 | 5.8E-95 | 148/165 (89%) | 156/165 (94%) |
|  |  |  | ENSG00000010810 |  |  |  |  |  |  |  |
|  |  |  | Z97989.1.1.155937 |  |  |  |  |  |  |  |
|  |  |  |  |  |  |  |  |  |  |  |
|  | Mouse | pep | ENSMUSP00000019988 | 39142277 | novel | MMU10 | 792 | 7.6E-96 | 149/165 (90%) | 156/165 (94%) |
|  |  |  | ENSMUSG00000019843 |  |  |  |  |  |  |  |
|  |  |  | CAAA01025685.1.1.49917 |  |  |  |  |  |  |  |
|  |  |  |  |  |  |  |  |  |  |  |
|  | Pufferfish | pep | SINFRUP00000149438 | 200674 | known |  | 789 | 1.6E-94 | 149/164 (90%) | 157/164 (95%) |
|  |  |  | SINFRUG00000140819 |  |  |  |  |  |  |  |
|  |  |  | scaffold_21 |  |  |  |  |  |  |  |
|  |  |  |  |  |  |  |  |  |  |  |
|  | Zebrafish | pep | ENSDARP00000003069 | 50381 | novel |  | 790 | 4.3E-95 | 149/164 (90%) | 158/164 (96%) |
|  |  |  | ENSDARG00000011370 |  |  |  |  |  |  |  |
|  |  |  | ctg24016.1 |  |  |  |  |  |  |  |
|  |  |  |  |  |  |  |  |  |  |  |
| OMM5003 | Human | gen | AL049634.8.1.115478 |  |  |  | 224 | 0.98 | 151/253 (59%) | 154/253 (60%) |
|  |  |  |  |  |  |  |  |  |  |  |
|  | Pufferfish | gen | scaffold_107 |  |  |  | 301 | 0.000067 | 121/176 (68%) | 121/176 (68%) |
|  |  |  |  |  |  |  |  |  |  |  |
| OMM5004 | Human | gen | AC011407.6.1.240957 |  |  |  | 237 | 0.073 | 143/234 (61%) | 143/234 (61%) |
|  |  |  |  |  |  |  |  |  |  |  |
| OMM5005 | Human | pep | ENSP00000242826 | 48284843 | known | HSA13 | 952 | 4.4E-96 | 171/196 (87%) | 186/196 (94%) |
|  |  |  | ENSG00000123178 |  |  |  |  |  |  |  |
|  |  |  | AL136123.19.1.178151 |  |  |  |  |  |  |  |
|  |  |  |  |  |  |  |  |  |  |  |
|  | Mouse | pep | ENSMUSP00000022497 | 52173296 | known | MMU14 | 954 | 3.2E-96 | 172/196 (87%) | 187/196 (95%) |
|  |  |  | ENSMUSG00000021930 |  |  |  |  |  |  |  |
|  |  |  | CAAA01047580.1.1.33856 |  |  |  |  |  |  |  |
|  |  |  |  |  |  |  |  |  |  |  |
|  | Pufferfish | pep | SINFRUP00000141669 | 7887 | known |  | 1003 | 2.3E-101 | 179/196 (91%) | 190/196 (96%) |
|  |  |  | SINFRUG00000133725 |  |  |  |  |  |  |  |
|  |  |  | scaffold_1057 |  |  |  |  |  |  |  |
|  |  |  |  |  |  |  |  |  |  |  |
|  | Zebrafish | pep | ENSDARP00000005279 | 113331 | novel |  | 994 | 1.5E-100 | 178/196 (90%) | 188/196 (95%) |
|  |  |  | ENSDARG00000012543 |  |  |  |  |  |  |  |
|  |  |  | ctg24123.1 |  |  |  |  |  |  |  |
|  |  |  |  |  |  |  |  |  |  |  |
| OMM5006 | Zebrafish | pep | ENSDARP00000018117 | 400441 | novel |  | 225 | 4.6E-19 | 43/50 (86%) | 47/50 (94%) |
|  |  |  | ENSDARG00000011308 |  |  |  |  |  |  |  |
|  |  |  | ctg10548.3 |  |  |  |  |  |  |  |
|  |  |  |  |  |  |  |  |  |  |  |
| OMM5008 | Human | gen | AL158073.10.1.186334 |  |  |  | 380 | 0.000000056 | 190/304 (62%) | 190/304 (62%) |
|  |  |  |  |  |  |  |  |  |  |  |
| OMM5015 | Zebrafish | pep | ENSDARP00000017273 | 31776 | novel |  | 211 | 1E-24 | 40/64 (62%) | 50/64 (78%) |
|  |  |  | ENSDARG00000017127 |  |  |  |  |  |  |  |
|  |  |  | ctg12225.1 |  |  |  |  |  |  |  |
|  |  |  |  |  |  |  |  |  |  |  |
| OMM5017 | Human | gen | AC080078.7.1.150567 |  |  |  | 209 | 0.000000033 | 61/79 (77%) | 61/79 (77%) |
|  |  |  |  |  |  |  |  |  |  |  |
|  | Mouse | gen | CAAA01011304.1.1.64834 |  |  | MMU3 | 203 | 0.000000075 | 61/79 (77%) | 61/79 (77%) |
|  |  |  |  |  |  |  |  |  |  |  |
| OMM5019 | Human | pep | ENSP00000333982 | 8539743 | known | HSA17 | 285 | 2.1E-25 | 62/99 (62%) | 73/99 (73%) |
|  |  |  | ENSG00000166579 |  |  |  |  |  |  |  |
|  |  |  | AC026130.15.1.182909 |  |  |  |  |  |  |  |
|  |  |  |  |  |  |  |  |  |  |  |
|  | Mouse | pep | ENSMUSP00000018880 | 69480796 | known | MMU11 | 251 | 9.9E-22 | 58/99 (58%) | 67/99 (67%) |
|  |  |  | ENSMUSG00000018736 |  |  |  |  |  |  |  |
|  |  |  | AL603662.11.1.201275 |  |  |  |  |  |  |  |
|  |  |  |  |  |  |  |  |  |  |  |
|  | Pufferfish | pep | SINFRUP00000158368 | 9753 | known |  | 290 | 8.3E-26 | 63/98 (64%) | 70/98 (71%) |
|  |  |  | SINFRUG00000148978 |  |  |  |  |  |  |  |
|  |  |  | scaffold_841 |  |  |  |  |  |  |  |
|  |  |  |  |  |  |  |  |  |  |  |
|  | Zebrafish | pep | ENSDARP00000003032 | 64144 | novel |  | 376 | 4.6E-35 | 77/99 (77%) | 84/99 (84%) |
|  |  |  | ENSDARG00000010953 |  |  |  |  |  |  |  |
|  |  |  | ctg9827.1 |  |  |  |  |  |  |  |
|  |  |  |  |  |  |  |  |  |  |  |
| OMM5024 | Human | gen | AP001153.5.1.170427 |  |  |  | 242 | 0.4 | 172/286 (60%) | 172/286 (60%) |
|  |  |  |  |  |  |  |  |  |  |  |
| OMM5030 | Human | gen | AC079462.2.1.77050 |  |  |  | 224 | 0.95 | 216/388 (55%) | 216/388 (55%) |
|  |  |  |  |  |  |  |  |  |  |  |
| OMM5034 | Zebrafish | gen | ctg14531.2 |  |  |  | 423 | 2.8E-18 | 129/168 (76%) | 129/168 (76%) |
|  |  |  |  |  |  |  |  |  |  |  |
|  |  |  |  |  |  |  |  |  |  |  |
| OMM5041 | Human | pep | ENSP00000308738 | 168722610 | known | HSA3 | 653 | 6.3E-73 | 129/149 (86%) | 142/149 (95%) |
|  |  |  | ENSG00000114209 |  |  |  |  |  |  |  |
|  |  |  | AC079822.13.1.181672 |  |  |  |  |  |  |  |
|  |  |  |  |  |  |  |  |  |  |  |
|  | Mouse | pep | ENSMUSP00000029424 | 76433096 | known | MMU3 | 653 | 7.5E-73 | 129/149 (86%) | 142/149 (95%) |
|  |  |  | ENSMUSG00000027835 |  |  |  |  |  |  |  |
|  |  |  | CAAA01216938.1.1.30576 |  |  |  |  |  |  |  |
|  |  |  |  |  |  |  |  |  |  |  |
|  | Pufferfish | pep | SINFRUP00000150477 | 76633 | known |  | 696 | 1.5E-77 | 139/149 (93%) | 147/149 (98%) |
|  |  |  | SINFRUG00000141769 |  |  |  |  |  |  |  |
|  |  |  | scaffold_946 |  |  |  |  |  |  |  |
|  |  |  |  |  |  |  |  |  |  |  |
|  | Zebrafish | pep | ENSDARP00000021450 | 738954 | novel |  | 661 | 1.8E-73 | 132/149 (88%) | 142/149 (95%) |
|  |  |  | ENSDARG00000012591 |  |  |  |  |  |  |  |
|  |  |  | ctg1311.4 |  |  |  |  |  |  |  |
|  |  |  |  |  |  |  |  |  |  |  |
| OMM5045 | Human | pep | ENSP00000266481 | 32723491 | known | HSA12 | 472 | 5.7E-45 | 99/137 (72%) | 113/137 (82%) |
|  |  |  | ENSG00000087470 |  |  |  |  |  |  |  |
|  |  |  | AC084824.20.1.98236 |  |  |  |  |  |  |  |
|  |  |  |  |  |  |  |  |  |  |  |
|  | Mouse | pep | ENSMUSP00000023477 | 15779025 | known | MMU16 | 472 | 8.7E-45 | 99/137 (72%) | 113/137 (82%) |
|  |  |  | ENSMUSG00000022789 |  |  |  |  |  |  |  |
|  |  |  | CAAA01188737.1.1.153834 |  |  |  |  |  |  |  |
|  |  |  |  |  |  |  |  |  |  |  |
|  | Pufferfish | pep | SINFRUP00000151018 | 32838 | known |  | 394 | 1.8E-36 | 85/120 (70%) | 95/120 (79%) |
|  |  |  | SINFRUG00000142259 |  |  |  |  |  |  |  |
|  |  |  | scaffold_1711 |  |  |  |  |  |  |  |
|  |  |  |  |  |  |  |  |  |  |  |
|  | Zebrafish | pep | ENSDARP00000006503 | 417189 | novel |  | 585 | 3.3E-57 | 121/139 (87%) | 127/139 (91%) |
|  |  |  | ENSDARG00000013904 |  |  |  |  |  |  |  |
|  |  |  | ctg9404.3 |  |  |  |  |  |  |  |
|  |  |  |  |  |  |  |  |  |  |  |
| OMM5051 | Human | cDNA | ENST00000234310 | 68380523 | known | HSA2 | 289 | 0.0000001 | 273/488 (55%) | 273/488 (55%) |
|  |  |  | ENSG00000115953 |  |  |  |  |  |  |  |
|  |  |  | AC017083.7.1.198278 |  |  |  |  |  |  |  |
|  |  |  |  |  |  |  |  |  |  |  |
| OMM5056 | Human | pep | ENSP00000325636 | 74736016 | known | HSA10 | 202 | 1.3E-16 | 47/124 (37%) | 70/124 (56%) |
|  |  |  | ENSG00000177791 |  |  |  |  |  |  |  |
|  |  |  | AC073389.10.1.190681 |  |  |  |  |  |  |  |
|  |  |  |  |  |  |  |  |  |  |  |
|  | Mouse | pep | ENSMUSP00000022357 | 15618351 | known | MMU14 | 217 | 4E-18 | 49/124 (39%) | 70/124 (56%) |
|  |  |  | ENSMUSG00000021818 |  |  |  |  |  |  |  |
|  |  |  | AC121599.3.1.218747 |  |  |  |  |  |  |  |
|  |  |  |  |  |  |  |  |  |  |  |
| OMM5059 | Human | pep | ENSP00000255317 | 29107669 | known | HSA13 | 432 | 5.6E-41 | 83/139 (59%) | 105/139 (75%) |
|  |  |  | ENSG00000132965 |  |  |  |  |  |  |  |
|  |  |  | AL512642.18.1.139255 |  |  |  |  |  |  |  |
|  |  |  |  |  |  |  |  |  |  |  |
|  | Mouse | pep | ENSMUSP00000031658 | 147098525 | known | MMU5 | 414 | 5.3E-39 | 80/139 (57%) | 100/139 (71%) |
|  |  |  | ENSMUSG00000029653 |  |  |  |  |  |  |  |
|  |  |  | CAAA01172107.1.1.7341 |  |  |  |  |  |  |  |
|  |  |  |  |  |  |  |  |  |  |  |
|  | Pufferfish | pep | SINFRUP00000163111 | 31838 | known |  | 335 | 1.4E-30 | 63/81 (77%) | 72/81 (88%) |
|  |  |  | SINFRUG00000153302 |  |  |  |  |  |  |  |
|  |  |  | scaffold_1771 |  |  |  |  |  |  |  |
|  |  |  |  |  |  |  |  |  |  |  |
| OMM5062 | Pufferfish | gen | scaffold_1104 |  |  |  | 449 | 0.000000000000096 | 145/205 (70%) | 145/205 (70%) |
|  |  |  |  |  |  |  |  |  |  |  |
| OMM5067 | Pufferfish | cDNA | SINFRUT00000137134 | 198068 | known |  | 237 | 0.0055 | 101/159 (63%) | 101/159 (63%) |
|  |  |  | SINFRUG00000129594 |  |  |  |  |  |  |  |
|  |  |  | scaffold_220 |  |  |  |  |  |  |  |
|  |  |  |  |  |  |  |  |  |  |  |
| OMM5075 | Pufferfish | cDNA | SINFRUT00000147229 | 157 | known |  | 340 | 0.00000003 | 120/176 (68%) | 120/176 (68%) |
|  |  |  | SINFRUG00000138802 |  |  |  |  |  |  |  |
|  |  |  | scaffold_4382 |  |  |  |  |  |  |  |
|  |  |  |  |  |  |  |  |  |  |  |
| OMM5077 | Mouse | pep | ENSMUSP00000026723 | 39557217 | known | MMUX | 184 | 0.000000000000013 | 33/35 (94%) | 34/35 (97%) |
|  |  |  | ENSMUSG00000025630 |  |  |  |  |  |  |  |
|  |  |  | CAAA01210585.1.1.36085 |  |  |  |  |  |  |  |
|  |  |  |  |  |  |  |  |  |  |  |
|  | Pufferfish | pep | SINFRUP00000137814 | 49867 | known |  | 191 | 2.6E-15 | 35/35 (100%) | 35/35 (100%) |
|  |  |  | SINFRUG00000130219 |  |  |  |  |  |  |  |
|  |  |  | scaffold_354 |  |  |  |  |  |  |  |
|  |  |  |  |  |  |  |  |  |  |  |
| OMM5093 | Human | pep | ENSP00000307059 | 37725535 | known | HSA4 | 241 | 3.5E-30 | 47/64 (73%) | 54/64 (84%) |
|  |  |  | ENSG00000169299 |  |  |  |  |  |  |  |
|  |  |  | AC021106.6.1.195108 |  |  |  |  |  |  |  |
|  |  |  |  |  |  |  |  |  |  |  |
|  | Mouse | pep | ENSMUSP00000031063 | 63112353 | known | MMU5 | 248 | 1.2E-30 | 49/64 (76%) | 53/64 (82%) |
|  |  |  | ENSMUSG00000029171 |  |  |  |  |  |  |  |
|  |  |  | CAAA01215931.1.1.25469 |  |  |  |  |  |  |  |
|  |  |  |  |  |  |  |  |  |  |  |
|  | Pufferfish | pep | SINFRUP00000132806 | 3712 | known |  | 261 | 4.3E-32 | 51/64 (79%) | 56/64 (87%) |
|  |  |  | SINFRUG00000125652 |  |  |  |  |  |  |  |
|  |  |  | scaffold_1598 |  |  |  |  |  |  |  |
|  |  |  |  |  |  |  |  |  |  |  |
|  | Zebrafish | pep | ENSDARP00000027790 | 732549 | novel |  | 266 | 2.6E-33 | 50/64 (78%) | 57/64 (89%) |
|  |  |  | ENSDARG00000018178 |  |  |  |  |  |  |  |
|  |  |  | ctg26375.4 |  |  |  |  |  |  |  |
|  |  |  |  |  |  |  |  |  |  |  |
| OMM5099 | Human | pep | ENSP00000311651 | 130810307 | known | HSA8 | 513 | 1.5E-49 | 98/108 (90%) | 106/108 (98%) |
|  |  |  | ENSG00000153310 |  |  |  |  |  |  |  |
|  |  |  | AC022973.5.1.194367 |  |  |  |  |  |  |  |
|  |  |  |  |  |  |  |  |  |  |  |
|  | Mouse | pep | ENSMUSP00000023010 | 64118423 | known | MMU15 | 510 | 3.6E-49 | 97/108 (89%) | 106/108 (98%) |
|  |  |  | ENSMUSG00000022378 |  |  |  |  |  |  |  |
|  |  |  | CAAA01197070.1.1.43158 |  |  |  |  |  |  |  |
|  |  |  |  |  |  |  |  |  |  |  |
|  | Pufferfish | pep | SINFRUP00000142542 | 378851 | known |  | 430 | 1.2E-40 | 83/88 (94%) | 86/88 (97%) |
|  |  |  | SINFRUG00000134531 |  |  |  |  |  |  |  |
|  |  |  | scaffold_4 |  |  |  |  |  |  |  |
|  |  |  |  |  |  |  |  |  |  |  |
|  | Zebrafish | pep | ENSDARP00000021229 | 393333 | novel |  | 491 | 3E-47 | 96/108 (88%) | 100/108 (92%) |
|  |  |  | ENSDARG00000020929 |  |  |  |  |  |  |  |
|  |  |  | ctg25820.3 |  |  |  |  |  |  |  |
|  |  |  |  |  |  |  |  |  |  |  |
| OMM5100 | Pufferfish | pep | SINFRUP00000160712 | 29893 | novel |  | 341 | 3.3E-31 | 79/140 (56%) | 100/140 (71%) |
|  |  |  | SINFRUG00000151144 |  |  |  |  |  |  |  |
|  |  |  | scaffold_272 |  |  |  |  |  |  |  |
|  |  |  |  |  |  |  |  |  |  |  |
|  | Zebrafish | pep | ENSDARP00000009326 | 1777 | novel |  | 333 | 1.6E-30 | 79/142 (55%) | 97/142 (68%) |
|  |  |  | ENSDARG00000011657 |  |  |  |  |  |  |  |
|  |  |  | ctg25901.1 |  |  |  |  |  |  |  |
|  |  |  |  |  |  |  |  |  |  |  |
| OMM5112 | Zebrafish | pep | ENSDARP00000021882 | 69187 | novel |  | 252 | 6.3E-22 | 53/81 (65%) | 59/81 (72%) |
|  |  |  | ENSDARG00000008621 |  |  |  |  |  |  |  |
|  |  |  | ctg12979.1 |  |  |  |  |  |  |  |
|  |  |  |  |  |  |  |  |  |  |  |
| OMM5113 | Human | cDNA | ENST00000027474 | 108237539 | known | HSA6 | 306 | 0.000000039 | 114/165 (69%) | 114/165 (69%) |
|  |  |  | ENSG00000025796 |  |  |  |  |  |  |  |
|  |  |  | AL024507.7.1.152408 |  |  |  |  |  |  |  |
|  |  |  |  |  |  |  |  |  |  |  |
|  | Mouse | cDNA | ENSMUST00000019937 | 42596987 | known | MMU10 | 315 | 0.000000016 | 115/165 (69%) | 115/165 (69%) |
|  |  |  | ENSMUSG00000019802 |  |  |  |  |  |  |  |
|  |  |  | CAAA01101521.1.1.43107 |  |  |  |  |  |  |  |
|  |  |  |  |  |  |  |  |  |  |  |
|  | Zebrafish | cDNA | ENSDART00000021035 | 558355 | novel |  | 425 | 0.000000000000087 | 129/169 (76%) | 129/169 (76%) |
|  |  |  | ENSDARG00000017740 |  |  |  |  |  |  |  |
|  |  |  | ctg10561.3 |  |  |  |  |  |  |  |
|  |  |  |  |  |  |  |  |  |  |  |
| OMM5117 | Human | pep | ENSP00000311913 | 88093005 | known | HSA10 | 334 | 3.7E-37 | 64/101 (63%) | 78/101 (77%) |
|  |  |  | ENSG00000122367 |  |  |  |  |  |  |  |
|  |  |  | AC067750.5.1.203205 |  |  |  |  |  |  |  |
|  |  |  |  |  |  |  |  |  |  |  |
|  | Mouse | pep | ENSMUSP00000022328 | 29262207 | known | MMU14 | 337 | 7.4E-32 | 65/101 (64%) | 78/101 (77%) |
|  |  |  | ENSMUSG00000021798 |  |  |  |  |  |  |  |
|  |  |  | CAAA01053558.1.1.13556 |  |  |  |  |  |  |  |
|  |  |  |  |  |  |  |  |  |  |  |
|  | Zebrafish | pep | ENSDARP00000002993 | 189931 | novel |  | 259 | 1.1E-22 | 50/78 (64%) | 63/78 (80%) |
|  |  |  | ENSDARG00000014248 |  |  |  |  |  |  |  |
|  |  |  | ctg10256.1 |  |  |  |  |  |  |  |
|  |  |  |  |  |  |  |  |  |  |  |
| OMM5121 | Mouse | pep | ENSMUSP00000031929 | 41298243 | known | MMU6 | 364 | 1.1E-33 | 63/120 (52%) | 83/120 (69%) |
|  |  |  | ENSMUSG00000029871 |  |  |  |  |  |  |  |
|  |  |  | CAAA01153920.1.1.10378 |  |  |  |  |  |  |  |
|  |  |  |  |  |  |  |  |  |  |  |
|  | Pufferfish | pep | SINFRUP00000148680 | 2472 | known |  | 404 | 6.9E-38 | 70/89 (78%) | 77/89 (86%) |
|  |  |  | SINFRUG00000140125 |  |  |  |  |  |  |  |
|  |  |  | scaffold_2683 |  |  |  |  |  |  |  |
|  |  |  |  |  |  |  |  |  |  |  |
| OMM5127 | Human | pep | ENSP00000263021 | 29869417 | known | HSA16 | 299 | 1E-25 | 62/98 (63%) | 75/98 (76%) |
|  |  |  | ENSG00000013364 |  |  |  |  |  |  |  |
|  |  |  | AC120114.2.1.140666 |  |  |  |  |  |  |  |
|  |  |  |  |  |  |  |  |  |  |  |
|  | Mouse | pep | ENSMUSP00000032919 | 115702812 | known | MMU7 | 300 | 8.6E-26 | 64/108 (59%) | 80/108 (74%) |
|  |  |  | ENSMUSG00000030681 |  |  |  |  |  |  |  |
|  |  |  | CAAA01013628.1.1.41423 |  |  |  |  |  |  |  |
|  |  |  |  |  |  |  |  |  |  |  |
|  | Zebrafish | gen | ctg9991.1 |  |  |  | 535 | 1.6E-28 | 151/197 (76%) | 151/197 (76%) |

^a^DB = database

^b^Chr = chromosome

^c^E-Value = expectation value
