# Supplementary material for: Comparative mapping of expressed sequence tags containing microsatellites in rainbow trout (Oncorhynchus mykiss)
Source: BMC Genomics. 2005 Apr 18;6:54. doi: 10.1186/1471-2164-6-54 (PMC1090573; doi:10.1186/1471-2164-6-54)
Supplement: Additional File 5 — Appendix 5. In silico derived comparative mapping information II. BLAST was used to identify similar sequences with tetraodon. [file 1471-2164-6-54-S5.doc]

Appendix 5. *In silico* derived comparative mapping information II. BLAST was used to identify similar sequences with tetraodon.

| Query Definition | Query Length | Hit Definition | Hit Length | Bit Score | Score | Expectation  Value | Query From | Query To | Hit From | Hit To | Query Frame | Hit Frame | Identity | Positive | Gaps | Align Length |
| --- | --- | --- | --- | --- | --- | --- | --- | --- | --- | --- | --- | --- | --- | --- | --- | --- |
| OMM5000 | 680 | chr8 | 10308592 | 184.852 | 93 | 3.58E-45 | 474 | 198 | 8479766 | 8480043 | 1 | -1 | 236 | 236 | 1 | 278 |
| OMM5002 | 1227 | chr21 | 5821691 | 137.276 | 69 | 1.37E-30 | 268 | 144 | 1130175 | 1130299 | 1 | -1 | 111 | 111 |  | 125 |
| OMM5002 | 1227 | chr21 | 5821691 | 105.558 | 53 | 4.84E-21 | 402 | 266 | 1129894 | 1130030 | 1 | -1 | 116 | 116 |  | 137 |
| OMM5002 | 1227 | chr21 | 5821691 | 97.6284 | 49 | 1.18E-18 | 609 | 402 | 1129569 | 1129776 | 1 | -1 | 168 | 168 |  | 208 |
| OMM5002 | 1227 | chr10 | 12859810 | 103.575 | 52 | 1.91E-20 | 117 | 268 | 8206698 | 8206849 | 1 | 1 | 127 | 127 |  | 152 |
| OMM5002 | 1227 | chr10 | 12859810 | 87.7166 | 44 | 1.14E-15 | 177 | 268 | 8224063 | 8224154 | 1 | 1 | 80 | 80 |  | 92 |
| OMM5002 | 1227 | chr6 | 6216017 | 81.7695 | 41 | 7.02E-14 | 268 | 144 | 4456046 | 4456170 | 1 | -1 | 104 | 104 |  | 125 |
| OMM5005 | 1037 | chr2 | 19247847 | 101.728 | 51 | 5.81E-20 | 496 | 594 | 8693410 | 8693508 | 1 | 1 | 87 | 87 |  | 99 |
| OMM5005 | 1037 | chr2 | 19247847 | 101.728 | 51 | 5.81E-20 | 1 | 95 | 8691028 | 8691122 | 1 | 1 | 84 | 84 |  | 95 |
| OMM5005 | 1037 | chr2 | 19247847 | 83.8627 | 42 | 1.39E-14 | 109 | 218 | 8691933 | 8692042 | 1 | 1 | 93 | 93 |  | 110 |
| OMM5034 | 676 | chr6 | 6216017 | 99.6107 | 50 | 1.63E-19 | 95 | 30 | 4984393 | 4984458 | 1 | -1 | 62 | 62 |  | 66 |
| OMM5041 | 670 | chr10 | 12859810 | 107.54 | 54 | 6.61E-22 | 522 | 417 | 8650279 | 8650384 | 1 | -1 | 93 | 93 |  | 106 |
| OMM5041 | 670 | chr10 | 12859810 | 101.593 | 51 | 4.08E-20 | 229 | 123 | 8652270 | 8652376 | 1 | -1 | 93 | 93 |  | 107 |
| OMM5041 | 670 | chr10 | 12859810 | 71.8577 | 36 | 3.64E-11 | 401 | 282 | 8651021 | 8651140 | 1 | -1 | 99 | 99 |  | 120 |
| OMM5045 | 701 | chr12 | 12535257 | 83.7519 | 42 | 1E-14 | 344 | 215 | 658033 | 658162 | 1 | -1 | 108 | 108 |  | 130 |
| OMM5064 | 694 | chr21 | 5821691 | 73.8401 | 37 | 9.56E-12 | 476 | 408 | 753391 | 753459 | 1 | -1 | 61 | 61 |  | 69 |
| OMM5098 | 667 | chr3 | 14679700 | 109.523 | 55 | 1.66E-22 | 81 | 3 | 6184576 | 6184654 | 1 | -1 | 73 | 73 |  | 79 |
| OMM5099 | 685 | chr6 | 6216017 | 125.381 | 63 | 2.88E-27 | 188 | 66 | 4789390 | 4789512 | 1 | -1 | 108 | 108 |  | 123 |
| OMM5099 | 685 | chr6 | 6216017 | 75.8225 | 38 | 2.39E-12 | 66 | 1 | 4789578 | 4789643 | 1 | -1 | 59 | 59 |  | 66 |
| OMM5099 | 685 | chr21 | 5821691 | 77.8048 | 39 | 6.04E-13 | 191 | 121 | 4228297 | 4228367 | 1 | -1 | 63 | 63 |  | 71 |
| OMM5100 | 703 | chr19 | 5206847 | 107.54 | 54 | 6.94E-22 | 309 | 410 | 1693253 | 1693354 | 1 | 1 | 90 | 90 |  | 102 |
| OMM5107 | 648 | chr9 | 10398049 | 89.699 | 45 | 1.5E-16 | 1 | 53 | 4010432 | 4010484 | 1 | 1 | 51 | 51 |  | 53 |
